# Evaluation of the degree of agreement in the diagnosis of diabetic retinopathy between ophthalmologists and EyeArt^®^

**DOI:** 10.1186/s40942-025-00748-4

**Published:** 2025-11-19

**Authors:** Isabel Inmaculada Guedes Guedes, Pedro Saavedra Santana, Francisco Cabrera López, Ángel Ramos Macías, Ángel Ramos de Miguel, Ayoze González Hernández

**Affiliations:** 1 Ophthalmology, Complejo Hospitalario Universitario Insular Materno Infantil de Gran Canaria, University of Las Palmas de Gran Canaria (ULPGC), Avda Marítima Del sur s/n. 35016, Las Palmas de Gran Canaria, Las Palmas Spain; 2 Department of Mathematics, University of Las Palmas de Gran Canaria (ULPGC), Las Palmas de Gran Canaria, Spain; 3 Otolaryngology, Complejo Hospitalario Universitario Insular Materno Infantil de Gran Canaria, University of Las Palmas de Gran Canaria (ULPGC), Las Palmas de Gran Canaria, Spain; 4 Department of Otolaryngology and Head and Neck Surgery University of Las Palmas de Gran Canaria (ULPGC), Las Palmas de Gran Canaria, Spain; 5 Neurology, Hospital Universitario de Gran Canaria Doctor Negrín, Fernando Pessoa University, Las Palmas de Gran Canaria, Spain

**Keywords:** Diabetic retinopathy, Artificial intelligence, Automated diabetic retinopathy, Artificial intelligence detection of diabetic retinopathy, Automated retinal image

## Abstract

**Objective or purpose:**

To evaluate the diagnostic performance and agreement of the EyeArt^®^ Artificial Intelligence (AI) system for detecting Diabetic Retinopathy (DR), comparing its results with ophthalmologists’ assessments in a regional screening program.

**Design:**

Cross-sectional observational study.

**Subjects, participants, and/or controls:**

A total of 498 diabetic patients aged 18 years or older were enrolled between June and September 2023 through the Retisalud DR screening program in the Canary Islands. No separate control group was included.

**Methods:**

All participants underwent non-mydriatic fundus photography using the TRC-NW400 camera. Retinal images were analyzed by the EyeArt^®^ AI system (version 2.1.0), and results were compared with assessments by ophthalmologists based on the International Clinical Diabetic Retinopathy scale (ICDR). Agreement was quantified using Cohen’s kappa coefficient. Additionally, mixed-effects logistic regression was used to explore associations between DR and clinical risk factors.

**Main outcome measures:**

Sensitivity, specificity, and agreement (Cohen’s kappa) of the AI system compared to clinical diagnosis; predictors of DR such as age, diabetes duration, presence of Diabetic Macular Edema (DME), and central retinal thickness (CRT-OCT).

**Results:**

The EyeArt® system achieved a binocular sensitivity of 100% (95% CI: 98.1–100) and a specificity of 93.5% (95% CI: 90.2–96.0). Agreement with ophthalmologist grading was excellent, with kappa values of 0.966 (right eye) and 0.978 (left eye). Younger age, longer diabetes duration, DME presence, and higher CRT were significantly associated with DR diagnosis.

**Conclusions:**

The EyeArt^®^ AI system showed excellent diagnostic accuracy and strong agreement with clinical evaluations in DR screening. Nonetheless, its tendency to overestimate DR severity indicates the need for further refinement of its grading algorithm. These findings support the potential integration of AI systems into large-scale DR screening programs, pending further validation.

**Supplementary information:**

The online version contains supplementary material available at 10.1186/s40942-025-00748-4.

## Introduction

Diabetic retinopathy (DR) is a prevalent microvascular complication of Diabetes Mellitus (DM), affecting an estimated one-third of all individuals with diabetes globally [1–3]. As the prevalence of diabetes continues to increase worldwide, the burden of DR has escalated, making it the leading cause of blindness among working-age adults [4]. In the United States alone, over 30 million adults live with DM, and studies indicate that more than 10% of these patients are at risk of vision-threatening DR (vtDR) if not monitored and treated promptly [1, 5]. Untreated DR progresses asymptomatically, advancing through stages marked by retinal damage, which can lead to irreversible vision loss if early signs are not detected and managed [6, 7].

The clinical importance of regular DR screening is underscored by the potential for early intervention to significantly reduce the progression of sight-threatening stages [8, 9]. Effective treatments, such as laser photocoagulation and intravitreal injections, have proven successful in halting or slowing the progression of DR [10–12]; however, their efficacy is maximized when applied at the onset of the disease [13]. Consequently, clinical guidelines recommend annual eye examinations for patients with DM to detect DR early [14, 15]. Yet, adherence to these recommendations is limited; in the United States, less than 60% of individuals with DM receive the recommended annual eye screening, with even lower rates in low-resource settings and among minority and underserved populations [16].

Traditional DR screening programs rely on human graders to manually assess digital fundus photographs [17, 18]. While effective, this approach is labor-intensive, expensive, and dependent on a limited pool of trained ophthalmic professionals [19]. In regions with high demand and limited resources, such as rural areas and developing countries, barriers such as lack of access to eye care specialists, lengthy wait times, and high costs further limit the reach and efficacy of DR screening efforts [20, 21]. Additionally, in low- and middle-income countries, the shortage of ophthalmologists and the logistical challenges of reaching remote populations exacerbate the gaps in screening and early detection [22, 23].

In response to these limitations, Artificial Intelligence (AI) has emerged as a promising tool to address the challenges of DR screening [24]. Recent advancements in AI-driven image analysis, powered by deep learning (DL) algorithms, allow automated detection of DR from retinal fundus photographs. AI-based screening systems, such as EyeArt^®^, IDx-DR^®^, and Retmarker^®^, have shown comparable accuracy to human graders in detecting referral-warranted DR (rwDR) and vtDR, offering high sensitivity and specificity rates that make them viable alternatives to traditional manual grading [25, 26]. These systems can be deployed in primary care and telemedicine settings, making DR screening more accessible and potentially reducing the overall healthcare costs associated with vision loss prevention [27, 28].

Among the available AI systems, EyeArt® was selected for this study due to practical considerations in our local healthcare context. Specifically, it was the system to which we had licensed access, allowing us to integrate it seamlessly into the existing Retisalud screening program infrastructure. Moreover, EyeArt® has demonstrated strong diagnostic performance in large population-based studies, further supporting its inclusion in this comparative analysis [29, 30].

One of the major advantages of AI-based DR screening is the ability to implement point-of-care testing in primary and community healthcare settings without the need for specialized equipment or training in ophthalmology [31, 32]. For example, studies have demonstrated that non-mydriatic cameras paired with AI algorithms can be operated effectively by general healthcare staff, facilitating DR screening in primary care practices and improving adherence to screening guidelines among underserved populations [33]. Furthermore, the integration of AI into telemedicine frameworks has enabled remote DR screening in rural and isolated areas, providing timely detection and referral recommendations without requiring patients to travel long distances to access specialized eye care [34, 35].

Economic evaluations of AI-based DR screening systems suggest that these tools can be cost-effective, especially when implemented at a large scale in settings with high diabetes prevalence [36]. Studies show that by reducing the need for manual grading and minimizing unnecessary referrals, AI-driven DR screening can lower healthcare costs while maintaining high diagnostic accuracy [26, 37–40]. Moreover, AI systems have the potential to alleviate the workload on human graders, enabling ophthalmologists to focus on cases that require immediate intervention and enhancing the overall efficiency of DR screening programs [41, 42].

The objective of this study is to evaluate the diagnostic performance of the EyeArt® AI system in detecting DR, by analyzing its concordance with ophthalmologist-determined diagnoses within a large-scale regional screening program. The study aims to determine the degree of agreement between both diagnostic approaches and assess the potential utility of EyeArt® as a reliable tool in early DR detection.

## Methods

### Design

This is a cross-sectional study in which 499 diabetic patients over 18 years old were included. Patients were randomly recruited from the DR screening program conducted in the Canary Islands (*Retisalud*) between June 1, 2023, and September 22, 2023. Once selected, they were scheduled for an appointment at the Ophthalmology Department of a tertiary-level hospital. During this consultation, patients were informed about the study, including the details of participation. All participants received detailed information about the study objectives and procedures and signed an informed consent form (Annex I) prior to enrollment. The study was reviewed and approved by the Ethics Committee of Las Palmas CEI/CEIm, under reference number 2022–376–1. All procedures complied with the Declaration of Helsinki and current data protection regulations.

In the recruitment consultation, in addition to demographic data such as age and sex, information was collected regarding the type of diabetes mellitus (type 1 or type 2) and associated conditions that increase cardiovascular risk. These conditions included hypertension, dyslipidemia, cardiac pathology, stroke, obstructive sleep apnea syndrome, and renal pathology.

Ophthalmological examination, included best-corrected visual acuity (BCVA), spherical equivalent (SE), crystalline lens status (assessing nuclear – Cristallyne N, cortical – Cristallyne C, and posterior subcapsular components – Criatallyne P), central retinal thickness measured by Optical Coherence Tomography (CRT- OCT) and vascular plexus density measured by angio-OCT. The OCT and OCT-angiography (OCT-A) images were obtained using the Triton OCT (Topcon^®^ Corporation, Japan). Following the examination and fundus evaluation, the degree of DR was determined by ophthalmologist based on the ICDR [43].

The EyeArt^®^ software generated a report determining the degree of DR and provided a specific score based on the severity of the condition. The characteristics of the sample included in the study are presented in Table 1.

**Table 1:** Patient characteristics: overall and by DR

	Overall *N* = 498	Diabetic Retinopathy			*P*-value
		No *N* = 309	Unilateral *N* = 70	Bilateral *N* = 119	
Age (years)	65.1 ± 11.1	65.8 ± 10.6	65.5 ± 11.6	63.0 ± 11.7	0.065
Sex male	266 (53.4)	159 (51.5)	37 (52.9)	70 (58.8)	0.39
Arterial hypertension	367 (73.7)	227 (73.5)	53 (75.7)	87 (73.1)	0.915
Type 2 diabetes mellitus	484 (97.2)	304 (98.4)	68 (97.1)	112 (94.1)	0.055
Dyslipidemia	236 (47.4)	146 (47.2)	37 (52.9)	53 (44.5)	0.541
Asthma	7 (1.4)	5 (1.6)	1 (1.4)	1 (0.8)	1
Acute myocardial infarction	16 (3.2)	11 (3.6)	1 (1.4)	4 (3.4)	0.869
COPD	25 (5.0)	15 (4.8)	3 (4.3)	7 (5.9)	0.834
IC	4 (0.8)	3 (1.0)	0	1 (0.8)	1
IRC	13 (2.6)	10 (3.2)	0	3 (2.5)	0.428
SAOS	6 (1.2)	5 (1.6)	0	1 (0.8)	0.849
Duration of DM (years)	8 (3; 10)	6 (2; 9)	9 (5; 10)	9 (6; 12)	< 0.001

Data are means ± SD, frequencies (%) and medians (IQR). AIM: Acute Myocardial Infarction. COPD: chronic obstructive pulmonary disease. HF: Heart failure. CKD: chronic kidney disease. OSAS: Obstructive sleep apnea syndrome

### Image acquisition and analysis

Eligible participants were screened using non-mydriatic fundus photographs taken with a TRC-NW400 (Topcon^®^ Corporation, Japan). The resultant images were subsequently analyzed using EyeArt 2.1.0 automated DR screening software (Eyenuk, Inc., Woodland Hills, CA). Images were evaluated for quality prior to analysis. Photographs with insufficient illumination, focus issues, or artifacts that impeded retinal evaluation were excluded from the final analysis. One participant was excluded due to poor image quality caused by advanced lens opacity (cataract), as the AI system could not process the data. The images were obtained under non-mydriatic conditions, using the same device and under identical lighting conditions throughout.

A total of 1,996 fundus images were captured (four per patient: one macula-centered and one optic disc-centered image for each eye). Image quality was assessed prior to analysis by both the ophthalmologists and the EyeArt® AI system. Of these, 1,992 images (99.8%) were deemed gradable and included in the final analysis, while 4 images (0.2%) were excluded due to media opacities (cataracts) that prevented adequate retinal visualization. Only one patient was completely excluded because both eyes were ungradable. These findings indicate an overall high image acquisition quality within the Retisalud screening workflow.

### Sample size and power calculation

The sample size was not determined based on a prior power analysis, but rather by operational limitations. A total of 500 patient licenses were made available by Eyenuk® (EyeArt®) for this research project, which defined the upper limit of included participants. Despite the absence of a formal power calculation, the sample of 498 patients provides a robust dataset for statistical modeling and concordance analysis.

### Statistical analysis

#### Univariate analysis

Categorical variables are expressed as frequencies and percentages and continuous as mean and standard deviation (SD) when data followed a normal distribution, or as median and interquartile range (IQR = 25^th^ − 75^th^ percentile) when distribution departed from normality. The percentages were compared, as appropriate, using the Chi-square (${\chi ^2}$) test or the exact Fisher test, the means by the t-test, and the medians by the Wilcoxon test for independent data.

#### Logistic mixed models for DR

To identify factors associated with DR for each diagnostic method (OFT and AI), mixed-effects logistic regression models were utilized, accounting for the nesting of observations for each eye within patients [44]. The mixed-effects logistic model is essential due to this nesting, which addresses intra-patient correlation between paired eyes. The models are formulated as:

$${\rm{logit}}\left\{ {{\rm{Pr}}\left( {DR = 1\mid {X_1},...,{X_p}} \right)} \right\} = {\beta _0} + \mathop \sum \limits_{j = 1}^p {\beta _j}{X_j} + u$$

Here, $DR$ is the binary outcome variable indicating the presence (1) or absence (0) of DR, ${X_1},...,{X_P}$ are the covariates and $u$ s the patient-specific random effect. We assume that the random effects are independent and normally distributed as $N\left( {0,{\sigma _u}} \right)$ random variables. The selection of covariates was based on the Akaike Information Criterion (AIC), which measures the relative lack of fit, penalizing for model complexity. A model ${M_1}$ is preferable to ${M_2}$ if $AIC\left( {{M_1}} \right) < AIC\left( {{M_2}} \right)$.

From the estimated models, we derived the intraclass correlation coefficient (ICC), which quantifies the correlation between observations from both eyes of the same patient. The ICC is defined as:

$$ICC = {{\sigma _u^2} \over {\sigma _u^2 + {\pi ^2}/3}}$$

where ${\pi ^2}/3 \approx 3.29$ approximates the residual variance from the underlying logistic distribution.

Models were estimated using maximum likelihood with the R package “glmmTMB” and summarized by $p$-values from likelihood ratio tests, changes in AIC values upon dropping each covariate, and odds ratios (OR) with 95% confidence intervals.

For each model (OFT and AI), a sensitivity analysis was performed by extracting 1000 random subsamples of size 300 each from the total sample (*n* = 498). With each of these samples, the logistic model for DR defined by OFT and AI was estimated. Robustness was visualized by comparing the OR of each of the factors across all samples, including the full sample.

Statistical significance was set at $P < 0.05$. Data were analyzed using the R package, version 4.2.1 [45].

#### Kappa statistic for agreement between diagnoses

To assess the agreement between the two diagnostic methods per eye, Cohen’s kappa [46] was estimated. This statistic is defined as:

$$\kappa = {{{P_o} - {P_e}} \over {1 - {P_e}}}$$

Here, ${P_o}$ and ${P_e}$ denote the proportions of observed agreements between the two diagnoses and those expected under the random agreement hypothesis.

For the sample size considered (*n* = 498), a *post-hoc* analysis was performed to determine the power of the study. The effect of the study was determined by means of the $h$ statistics, which is defined as [47]:

$$h = 2 \cdot \left( {arcsin\left( {\sqrt {{P_o}} } \right) - arcsin\left( {\sqrt {{P_e}} } \right)} \right)$$

The post-hoc power analysis was conducted using a one-sided test with a significance level of $\alpha = 0.05$ to determine if the observed agreement exceeded that expected by chance.

## Results

Four hundred ninety eight adults with diabetes consented to participate in this prospective study. One participant was excluded from the analysis because the fundus images obtained were of poor quality due to advanced cataracts and could not be analyzed by the AI program. Four hundred and ninety-eight adults with diabetes were ultimately included in the study.

Table 1 summarizes the patients characteristics, overall and DR (unilateral and bilateral). The mean age was 65.1 ± 11.1 of the patients were male, while 46.5% were female. The demographic analysis of the patients included in the study revealed no statistically significant differences concerning age, gender, type of DM, or the coexistence of other conditions that increase cardiovascular risk, such as hypertension, dyslipidemia, Obstructive Sleep Apnea Syndrome (OSAS), Chronic Obstructive Pulmonary Disease (COPD), asthma, heart failure, acute myocardial infarction, among others.

The Table 2 presents the clinical characteristics of right eyes of the sample and ophthalmological parameters obtained through OCT and other evaluation methods in the study sample, based on the degree of DR determined by the ophthalmologist. DME-OCT (Diabetic Macular Edema by OCT) showed statistically significant differences between groups (*p* < 0.001), indicating a higher presence of macular edema in more advanced stages of DR. Cortical Lens Status (Crystalline C) and posterior subcapsular component of the lens (Crystalline P) showed no significant differences between groups (*p* = 0.273 and *p* = 0.19 respectively), with most patients exhibiting no cortical and posterior subcapsular opacities. Nuclear Lens Status (Crystalline N) showed statistically significant differences (*p* = 0.019), reflecting increased nuclear lens opacification in more advanced stages of the disease. BCVA demonstrated a significant difference between groups (*p* = 0.023), suggesting a reduction in visual acuity in patients with more severe DR. Superficial Vascular Plexus Density (VD A-OCT) in the temporal and inferior regions exhibited significant differences (*p* < 0.001), indicating reduced vascular density associated with greater severity of DR. Other parameters, such as central and nasal superficial plexus density, did not show significant differences (*p* = 0.191 and *p* = 0.247, respectively). These results suggest a correlation between the severity of DR and structural and functional changes in the retina, particularly evident in visual acuity and temporal and inferior plexus vascular density.

**Table 2:** Right eyes. Summary of the characteristics of the right eyes of the patients included in the study

	Overall *N* = 498	Diabetic retinopathy diagnosed by OFT				*P*-value
		No *N* = 341	Mild non-proliferative *N* = 97	Moderate non-proliferative *N* = 45	Severe non-proliferative *N* = 15	
CRT-OCT	257 ± 38	255± 36	252 ± 32	277 ± 55	286 ± 30	< 0.001
DME-OCT	16 (3.2)	0	5 (5.2)	4 (8.9)	7 (46.7)	< 0.001
DME-AI	20 (4.0)	1 (0.3)	6 (6.2)	6 (13.3)	7 (46.7)	< 0.001
Crystalline.N	4 (2; 11)	3 (2; 6)	3 (1; 6)	4 (3; 11)	5 (3; 11)	0.019
Crystalline.C						0.273
0	465 (93.4)	317 (93.0)	94 (96.9)	41 (91.1)	13 (86.7)	
1	23 (4.6)	17 (5.0)	1 (1.0)	3 (6.7)	2 (13.3)	
2	8 (1.6)	6 (1.8)	1 (1.0)	1 (2.2)	0	
3	2 (0.4)	1 (0.3)	1 (1.0)	0	0	
Crystalline.P						0.19
0	491 (98.6)	336 (98.5)	95 (97.9)	45 (100)	15 (100)	
1	5 (1.0)	5 (1.5)	0	0	0	
2	2 (0.4)	0	2 (2.1)	0	0	
BCVA	0.80 (0.50; 0.80)	0.80 (0.50; 0.80)	0.80 (0.60; 0.90)	0.60 (0.40; 0.80)	0.70 (0.60; 0.80)	0.023
Spherical.equivalent	0.38 (−0.38; 1.38)	0.50 (−0.25; 1.50)	0.12 (−0.75; 1.00)	0.50 (−0.25; 1.75)	0 (−0.69; 0.38)	0.004
VD A-OCT OCT SUPERFICIAL PLEXUS CENTRAL	16 (13; 19)	16 (13; 19)	17 (13; 20)	16 (13; 19)	13 (11; 16)	0.191
VD A-OCT SUPERFICIAL PLEXUS SUPERIOR	46 (43; 48)	46 (43; 48)	45 (42; 47)	44 (42; 46)	43 (40; 46)	0.003
VD A-OCT SUPERFICIAL PLEXUS TEMPORAL	46 (44; 49)	47 (45; 49)	46 (44; 48)	45 (42; 47)	41 (40; 46)	< 0.001
VD A-OCT SUPERFICIAL PLEXUS INFERIOR	46 (42; 48)	47 (44; 48)	46 (42; 48)	43 (40; 45)	41 (40; 44)	< 0.001
VD A-OCT SUPERFICIAL PLEXUS.NASAL	45 (42; 47)	45 (43; 47)	44 (42; 47)	45 (42; 47)	43 (42; 45)	0.247

Data are frequencies (%) and medians (IQR). BCVA: best corrected visual acuity. Cristaline C: cortical component of the lens. Cristaline N: nuclear component of the lens. Cristaline P: posterior component of the lens. CRT OCT: central retinal thickness by optical coherence tomography. DME- AI: diabetic macular edema by artificial intelligence. DME-OCT: diabetic macular edema by optical coherence tomography. VD A-OCT: vascular density by angio-optical coherence tomography

Table 3 summarizes the characteristics of the left eyes of the patients included in the study, focusing on key clinical and ophthalmological parameters based on the degree of DR determined by the ophthalmologist. A statistically significant increase in the prevalence of macular edema (DME-OCT) was observed with disease severity (*p* < 0.001). Superficial vascular density (VD A-OCT) measurements revealed significant reductions in temporal plexus (*p* < 0.001). Additionally, CRT-OCT scores varied significantly across the stages of retinopathy, highlighting the systemic impact of DR on ocular health (*p* < 0.001). These results reinforce the importance of early detection and monitoring of macular edema, lens opacities, and vascular density changes in patients with DR.

**Table 3:** Left eyes. Summary of the characteristics of the left eyes of the patients included in the study

	Overall *N* = 498	Diabetic retinopathy diagnosed by OFT				*P-*value
		No *N* = 347	Mild non-proliferative *N* = 84	Moderate non-proliferative *N* = 47	Severe non-proliferative *N* = 20	
CRT-OCT	260.7 ± 36.3	255.5 ± 31.3	264.6 ± 34.1	278.0 ± 50.7	293.6 ± 53.7	< 0.001
DME-OCT	22 (4.4)	2 (0.6)	3 (3.6)	8 (17.0)	9 (45.0)	< 0.001
DME-AI	26 (5.2)	1 (0.3)	1 (1.2)	14 (29.8)	10 (50.0)	< 0.001
Crystalline.N	3 (2; 11)	3 (2; 6)	3 (1; 11)	4 (2; 11)	6 (3; 11)	0.081
Crystalline.C						0.559
0	465 (93.4)	324 (93.4)	77 (91.7)	46 (97.9)	18 (90.0)	
1	21 (4.2)	16 (4.6)	3 (3.6)	1 (2.1)	1 (5.0)	
2	11 (2.2)	6 (1.7)	4 (4.8)	0	1 (5.0)	
3	1 (0.2)	1 (0.3)	0	0	0	
Crystalline.P						0.663
0	495 (99.4)	345 (99.4)	83 (98.8)	47 (100.0)	20 (100.0)	
1	1 (0.2)	1 (0.3)	0	0	0	
2	1 (0.2)	0	1 (1.2)	0	0	
BCVA	1 (0.2)	1 (0.3)	0	0	0	
Spherical.equivalent	0.80 (0.60; 0.90)	0.80 (0.60; 0.95)	0.80 (0.60; 1.00)	0.70 (0.60; 0.80)	0.70 (0.50; 0.80)	0.073
VD A-OCT OCT SUPERFICIAL PLEXUS CENTRAL	0.50 (−0.25; 1.62)	0.62 (−0.12; 1.75)	0.00 (−1.12; 1.12)	0.25 (−0.75; 0.88)	0.31 (−0.16; 0.81)	0.001
VD A-OCT SUPERFICIAL PLEXUS SUPERIOR	16 (13; 19)	16 (13; 20)	17 (15; 20)	16 (12; 19)	14 (14; 19)	0.414
VD A-OCT SUPERFICIAL PLEXUS TEMPORAL	46 (43; 48)	47 (44; 48)	47 (43; 49)	44 (42; 46)	44 (41; 46)	< 0.001
VD A-OCT SUPERFICIAL PLEXUS INFERIOR	46 (44; 49)	47 (45; 49)	47 (44; 48)	45 (43; 47)	45 (41; 47)	0.012
VD A-OCT SUPERFICIAL PLEXUS.NASAL	46 (43; 48)	46 (43; 48)	45 (42; 47)	45 (42; 48)	45 (43; 47)	0.095
CRT-OCT	45 (42; 47)	45 (43; 47)	45 (42; 47)	43 (40; 45)	42 (40; 45)	0.001

Data are frequencies (%) and medians (IQR). BCVA: best corrected visual acuity. Cristaline C: cortical component of the lens. Cristaline N: nuclear component of the lens. Cristaline P: posterior component of the lens. crt oct: central retinal thickness by optical coherence tomography. DME- AI: diabetic macular edema by artificial intelligence. DME-OCT: diabetic macular edema by optical coherence tomography. VD A-OCT: vascular density by angio-optical coherence tomography

Table 4 provides a summary of the clinical and ophthalmological characteristics of the right eyes of the patients included in this study, based on the degree of DR determined by the AI. The results reveal significant differences in several parameters according to the severity of DR, while other variables remained unchanged. Regarding DME (DME - OCT), a statistically significant difference was observed between groups (*p* < 0.001), with a higher prevalence of macular edema as the severity of DR increased. The highest prevalence, 17.0%, was found in severe non-proliferative cases. In contrast, cortical lens opacity (Crystalline C) did not show significant differences between groups (*p* = 0.861). Most patients (93.4%) exhibited no cortical opacities, while a minority presented mild or moderate changes. Nuclear lens opacity (Crystalline N) exhibited a statistically significant increase in patients with greater disease severity (*p* = 0.011). Superficial vascular density (VD A-OCT) showed a significant reduction in the temporal, superior, inferior and nasal regions as the severity of DR increases. However, no significant differences were identified in the central region (*p* = 0.34)

**Table 4:** Right eyes. Summary of the characteristics of the left eyes of the patients included in the study

	Overall *N* = 498	Diabetic retinopathy diagnosed by AI				P-value
		No *N* = 324	Mild non- proliferative *N* = 57	Moderate non-proliferative *N* = 70	Severe non-proliferative/ Proliferative* N = 47	
CRT-OCT	257.1 ± 38.1	255.3 ± 36.4	249.6 ± 28.4	258.0 ± 50.2	277.1 ± 33.2	0.001
DME-OCT	16 (3.2)	0	0	8 (11.4)	8 (17.0)	< 0.001
DME-AI	20 (4.0)	0	0	9 (12.9)	11 (23.4)	< 0.001
Crystalline.N	4 (2; 11)	3 (2; 6)	3 (2; 6)	4 (1; 5)	5 (3; 11)	0.011
Crystalline.C						0.861
0	465 (93.4)	301 (92.9)	55 (96.5)	65 (92.9)	44 (93.6)	
1	23 (4.6)	17 (5.2)	1 (1.8)	3 (4.3)	2 (4.3)	
2	8 (1.6)	5 (1.5)	1 (1.8)	1 (1.4)	1 (2.1)	
3	2 (0.4)	1 (0.3)	0	1 (1.4)	0	
Crystalline.P						0.221
0	491 (98.6)	319 (98.5)	56 (98.2)	69 (98.6)	47 (100.0)	
1	5 (1.0)	5 (1.5)	0	0	0	
2	2 (0.4)	0	1 (1.8)	1 (1.4)	0	
BCVA	0.80 (0.50; 0.80)	0.80 (0.60; 0.80)	0.80 (0.50; 0.80)	0.80 (0.50; 0.88)	0.60 (0.50; 0.80)	0.405
Spherical.equivalent	0.38 (−0.38; 1.38)	0.50 (−0.25; 1.62)	0.00 (−1.38; 0.88)	0.12 (−0.59; 1.12)	0.38 (−0.56; 1.25)	0.036
VD A-OCT OCT SUPERFICIAL PLEXUS CENTRAL	16 (13; 19)	16 (13; 19)	16 (13; 20)	16 (13; 20)	15 (11; 18)	0.34
VD A-OCT SUPERFICIAL PLEXUS SUPERIOR	46 (43; 48)	46 (43; 48)	46 (43; 48)	45 (41; 47)	44 (40; 46)	< 0.001
VD A-OCT SUPERFICIAL PLEXUS TEMPORAL	46 (44; 49)	47 (45; 49)	46 (45; 49)	45 (43; 48)	44 (40; 46)	< 0.001
VD A-OCT SUPERFICIAL PLEXUS INFERIOR	46 (42; 48)	47 (44; 48)	47 (44; 48)	45 (40; 47)	42 (39; 44)	< 0.001
VD A-OCT SUPERFICIAL PLEXUS.NASAL	45 (42; 47)	45 (43; 47)	45 (42; 47)	44 (41; 47)	43 (41; 45)	0.009

Data are frequencies (%) and medians (IQR). BCVA: best corrected visual acuity. Cristaline C: cortical component of the lens. Cristaline N: nuclear component of the lens. Cristaline P: posterior component of the lens. CRT - OCT: central retinal thickness by optical coherence tomography. DME- AI: diabetic macular edema by artificial intelligence. DME - OCT: diabetic macular edema by optical coherence tomography. VD A-OCT: vascular density by angio-optical coherence tomography

The Table 5 provides a detailed breakdown of the clinical and ophthalmological characteristics of the patients’ eyes based on the degree of DR determined by the AI. Statistically significant differences were observed for DME (DME - OCT) across groups (*p* < 0.001), with a progressive increase in edema correlating strongly with the severity of DR. For lens opacities, no significant differences were found for cortical, nuclear and posterior opacities. BCVA declined significantly as DR severity increased (*p* = 0.004), highlighting the functional impact of disease progression. Regarding vascular density in the superficial plexus (VD A-OCT), the temporal and inferior regions exhibited a significant reduction in density with disease progression, indicating its association with advanced retinopathy.

**Table 5:** Left eyes. Summary of the characteristics of the left eyes of the patients included in the study

	Overall *N* = 498	Diabetic retinopathy diagnosed by AI				P-value
		No *N* = 336	Mild non-proliferative *N* = 51	Moderate non-proliferative *N* = 65	Severe non- proliferative/proliferative *N* = 46	
CRT-OCT	260.7 ± 36.3	255.7 ± 31.7	258.5 ± 25.7	271.9 ± 46.7	283.5 ± 48.4	< 0.001
DME-OCT	22 (4.4)	1 (0.3)	1 (2.0)	7 (10.8)	13 (28.3)	< 0.001
DME-AI	26 (5.2)	0	0	11 (16.9)	15 (32.6)	< 0.001
Crystalline.N	3 (2; 11)	3 (2; 6)	4 (1; 8)	4 (1; 11)	4 (2; 11)	0.126
Crystalline.C						0.541
0	465 (93.4)	314 (93.5)	49 (96.1)	58 (89.2)	44 (95.7)	
1	21 (4.2)	16 (4.8)	1 (2.0)	3 (4.6)	1 (2.2)	
2	11 (2.2)	5 (1.5)	1 (2.0)	4 (6.2)	1 (2.2)	
3	1 (0.2)	1 (0.3)	0	0	0	
Crystalline.P						0.694
0	495 (99.4)	334 (99.4)	51 (100.0)	64 (98.5)	46 (100.0)	
1	1 (0.2)	1 (0.3)	0	0	0	
2	1 (0.2)	0	0	1 (1.5)	0	
BCVA	1 (0.2)	1 (0.3)	0	0	0	0.004
Spherical.equivalent	0.8 (0.6; 0.9)	0.8 (0.6; 0.9)	0.8 (0.6; 1)	0.8 (0.5; 1)	0.6 (0.5; 0.8)	0.018
VD A-OCT OCT SUPERFICIAL PLEXUS CENTRAL	0.5 (−0.25; 1.62)	0.62 (−0.12; 1.75)	0 (−2; 0.88)	0.25 (−0.75; 1)	0.31 (−0.38; 0.88)	0.001
VD A-OCT SUPERFICIAL PLEXUS SUPERIOR	16 (13; 19)	16 (13; 20)	17 (15; 20)	16 (13; 19)	17 (13; 19)	0.571
VD A-OCT SUPERFICIAL PLEXUS TEMPORAL	46 (43; 48)	47 (44; 48)	47 (44; 50)	45 (42; 48)	43 (42; 46)	< 0.001
VD A-OCT SUPERFICIAL PLEXUS INFERIOR	46 (44; 49)	47 (45; 49)	47 (45; 49)	46 (43; 48)	45 (42; 47)	0.001
VD A-OCT SUPERFICIAL PLEXUS.NASAL	46 (43; 48)	46 (43; 48)	45 (42; 47)	45 (42; 47)	45 (42; 47)	0.042
CRT-OCT	45 (42; 47)	45 (43; 47)	45 (42; 47)	44 (40; 46)	42 (40; 44)	< 0.001

Data are frequencies (%) and medians (IQR). BCVA: best corrected visual acuity. Cristaline C: cortical component of the lens. Cristaline N: nuclear component of the lens. Cristaline P: posterior component of the lens. CRT - OCT: central retinal thickness by optical coherence tomography. DME - AI: diabetic macular edema by artificial intelligence. DME-OCT: diabetic macular edema by optical coherence tomography. VD A-OCT: vascular density by angio-optical coherence tomography

**Fig. 1:**
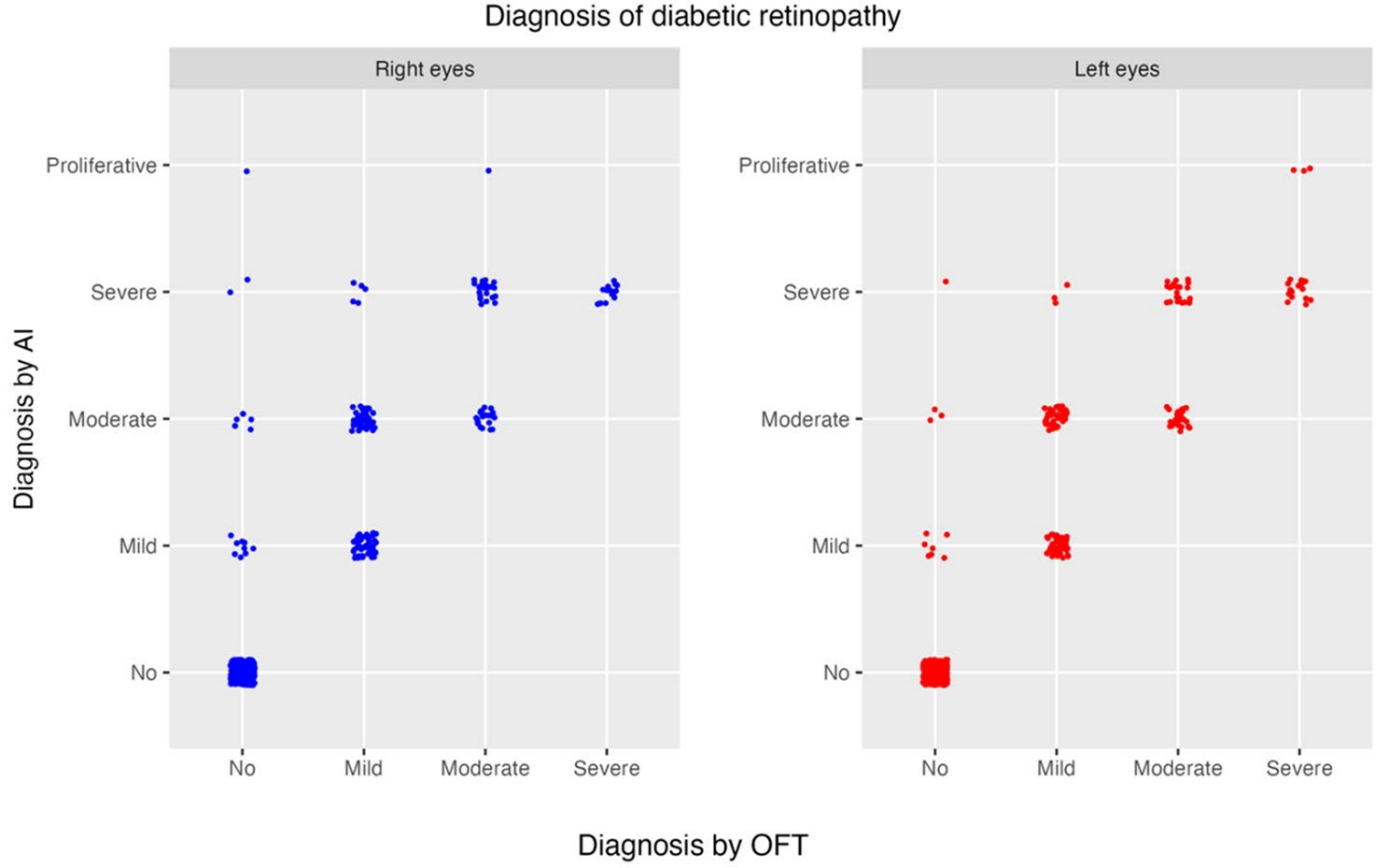
Scatter plots. Agreement between both diagnoses. The dots have been fluctuated to improve the impact of the frequencies

A stratified analysis of AI performance across different DR severity levels revealed that EyeArt^®^ tends to overestimate the disease severity, as demonstrated by the distribution of cases along the diagonal and above-diagonal in the scatter plot (Fig. 1). The proportion of moderate and severe classifications made by AI was higher than those assigned by ophthalmologists, suggesting a shift toward conservative (over-referral) predictions. This trend was more pronounced in the moderate non-proliferative and severe non-proliferative categories, whereas underestimation of severity was not observed. These findings support the earlier observation that the binary classification (presence/absence of DR) yielded better agreement metrics compared to the multi-level severity classification, as evidenced by the lower kappa coefficients in the latter.

Table 6 presents the results of a multivariable logistic regression model evaluating factors associated with DR. Age (per additional year) showed an inverse association with DR, with an OR = 0.976 (95% CI: 0.963–0.990; *p* < 0.001). This indicates that for each additional year of life, the odds of developing retinopathy decrease by approximately 2.4%, assuming that all other variables remain constant. This finding may reflect that disease progression is influenced by factors related to the duration of diabetes in individuals diagnosed at younger ages. In other words, individuals diagnosed at older ages have a lower risk of developing complications associated with DR because they have fewer years to develop them.

**Table 6:** Multivariate logistic regression for diabetic retinopathy

	Diabetic retinopathy			
	Diagnosed by OFT		Diagnosed by AI	
	P-value	Odd-Ratio (95% CI)	P-value	Odd-Ratio (95% CI)
Age, per year	< 0.001	0.976 (0.963; 0.990)	-	-
Years DM, per year	< 0.001	1.151 (1.109; 1.194)	< 0.001	1.120 (1.081; 1.160)
Type-1 diabetes mellitus			0.009	3.168 (1.339; 7.494)
DME - OCT*	< 0.001	32 (7.26; 142)	< 0.001	62.7 (8.38; 469)
CRT - OCT*	0.004	1.006 (1.002; 1.011)	0.047	1.004 (1.000; 1.009)
Spherical equivalent*	< 0.001	0.891 (0.832; 0.954)	< 0.001	0.880 (0.823; 0.942)
VD_A.OCT.SUPERFICIAL* PLEXUS.TEMPORAL	< 0.001	0.951 (0.925; 0.978)	0.001	0.957 (0.932; 0.983)

(*) per unit. CRT- OCT: central retinal thickness by optical coherence tomography. DME - OCT: diabetic macular edema by optical coherence tomography

The duration of diabetes showed a positive and significant association with DR. The OR = 1.151 (95% CI: 1.109–1.194; *p* < 0.001). This indicates that for each additional year since diabetes diagnosis, the odds of developing DR increase by 15.1%, while keeping other variables in the model constant. This result highlights the cumulative impact of diabetes on the development of microvascular complications over time. Another statistically significant determinant was the presence of DME determined by OCT, as its presence increased the probability of developing DR by 32 times (*p* < 0.001), assuming all other variables remain constant. Additionally, spherical equivalent demonstrated a statistically significant association with the development of DR (*p* < 0.001), acting as a protective factor in this case. Specifically, for each unit increase in spherical equivalent, the probability of developing DR decreased by 10.9%.

Table 7 presents the estimates of bSen (95% CI) and bSp (95% CI). According to Perera et al. [48], we will conduct the evaluation of AI as a diagnostic marker for DR using the concepts of binocular sensitivity (bSen) and binocular specificity (bSp). bSen is the probability of obtaining at least one positive AI diagnosis in one eye, given that there is a diagnosis of DR by an ophthalmologist (OFT) in at least one eye. bSp is the probability of obtaining a negative AI diagnosis in both eyes, given that there is a negative DR diagnosis by an ophthalmologist in both eyes.

**Table 7:** Sensitiviy and specificity(*) the ai is considered positive (+) when it is positive in at least one eye

AI	Diagnosis of DR in at least one eye		Binocular sensitivity and specificity (%)	
	Yes	No	*bSen* **[CI − 95%]**	*bSp* **[CI − 95%]**
(+) *	189	20	100 [98,1–100]	93,5 [90,2–96,0]
(-)	0	289		

The results presented in Table 7 indicate that the AI system demonstrated excellent diagnostic performance in detecting DR. The estimated binocular sensitivity (bSen) was 100% (95% CI: 98.1–100), meaning that the AI correctly identified all cases of DR in at least one eye, with no false negatives. This exceptionally high sensitivity suggests that the system is highly effective for screening purposes. The estimated binocular specificity (bSp) was 93.5% (95% CI: 90.2–96.0), indicating that the AI correctly classified the absence of DR in both eyes in 93.5% of negative cases, with a false positive rate of 6.5%.

Table 8 summarizes the levels of agreement for a binary (Yes/No) diagnosis, including the observed and expected proportions of agreement, as well as the kappa coefficients with their 95% CI. For the right eye, the observed agreement proportion was 0.966, while the expected agreement under the random hypothesis was 0.556, resulting in a kappa coefficient of 0.923 (95% CI: (0.887; 0.959). For the left eye, the observed agreement proportion was 0.978, with an expected agreement of 0.569, yielding a kappa coefficient of 0.949 (95% CI: (0.919; 0.979). These findings demonstrate almost perfect agreement for both eyes in the binary diagnosis.

**Table 8:** Agreement between binary (Y/N) diagnosis

	Proportion of agreements		*kappa* (95%CI)
	Observed	Expected*	
Right eye	0.966	0.556	0.923 (0.887; 0.959)
Left eye	0.978	0.569	0.949 (0.919; 0.979)

(*) Under the hypothesis of no agreement

Although the kappa coefficients indicate substantial to almost perfect agreement between AI and ophthalmologist diagnoses, particularly in binary classification.

### Kappa statistics for the agreement between diagnosis

The agreement between the two diagnostic methods, assessed per eye using Cohen’s kappa statistic, is presented in Table 8 (for the presence or absence of retinopathy) and Table 9 (for all retinopathy stages). For the right eye, the estimated kappa was 0.65; 95% CI = 0.594–0.707, while for the left eye it was 0.693; 95% CI = 0.636–0.75.

**Table 9:** Agreement between diagnosis

	Proportion of agreements		*kappa (95%CI)*	*h-statistics*	Power**
	Observed	Expected*			
Right eye	0.819	0.483	0.650 (0.594; 0.707)	0.726	1
Left eye	0.847	0.503	0.693 (0.636; 0.750)	0.761	1

(*) Under the hypothesis of no agreement

(**) For *n* = 498 and *α*=0.05

The post-hoc analysis yielded h statistic values of 0.726 for the right eye and 0.761 for the left eye. With a sample size of 498 and a significance level of 5%, this analysis, using a one-tailed test, demonstrated a power approaching unity for both eyes. This robust statistical power confirms the reliability of the study’s findings.

### Logistic mixed-models for the DR

Table 10 reports the mixed-effects logistic regression models for the presence or absence of DR, based on the OFT and AI diagnoses, respectively. For each variable included in the model, the table also provides the AIC values corresponding to the model obtained after removing that variable.

**Table 10:** Mixed models for *diabetic retinopathy*

	Diagnosis by OFT			Diagnosis by AI		
	*P*-value*	AIC**	Odd-Ratio (95% CI)	*P*-value*	AIC**	Odd-Ratio (95% CI)
*Age* (per year)	0.011	961.5	0.921 (0.868 - 0.978)	0.006	1051.4	0.946 (0.908 - 0.986)
Duration of DM (years)	< 0.001	970.7	1.585 (1.352 - 1.858)	< 0.001	1088.5	1.404 (1.233 - 1.598)
*GCR-OCT*	0.058	958.5	1.013 (1.001 - 1.026)	0.048	1047.6	1.009 (1 - 1.019)
*Spherical equivalent*	0.044	959	0.839 (0.710 - 0.992)	0.007	1050.9	0.834 (0.722 - 0.963)
*VD A. OCT superficial plexus temporal*	0.081	958	0.928 (0.862 - 0.999)	0.022	1049	0.933 (0.880 - 0.990)

(*) Likelihood ratio test

(**) The Akaike Information Criterion (AIC) measures relative model fit by balancing goodness-of-fit against model complexity; lower values indicate a superior trade-off. Removing a covariate typically increases the AIC, signaling reduced model quality. Model comparisons thus favor lower AIC values. For the full model based on OFT diagnosis, AIC = 957; for the full model based on AI, AIC = 1045.7. Notably, these AIC values for the full models are lower than those obtained upon covariate removal

The Fig. 1 consists of two scatter plots illustrating AI and ophthalmologist diagnostic outcomes for right eyes (blue) and left eyes (red).

X-axis (horizontal) represents the diagnosis performed by the ophthalmologist categorized as “No,” “Mild,” “Moderate,” and “Severe.” Y-axis (vertical) indicates the AI-generated diagnoses, classified using the same categories: “No,” “Mild,” “Moderate,” “Severe,” and an additional category, “Proliferative.” Each data point corresponds to a single AI prediction compared to its ground-truth diagnosis performed by the ophthalmologist. Right eye diagnoses are plotted in blue on the left panel and left eye diagnoses are plotted in red on the right panel. Points aligned along the diagonal (extending from the bottom-left to the top-right) indicate accurate AI predictions, where the AI diagnosis matches the diagnosis performed by the ophthalmologist. Points above the diagonal indicate instances where the AI overestimates the disease severity and points below the diagonal reflect cases where the AI underestimates the severity of the condition.

The scatter plots for right and left eyes exhibit similar distributions of data points, suggesting consistent diagnostic performance by the AI across both eyes. Both panels demonstrate a higher concentration of points in the “No” and “Mild” categories, indicating a predominance of low-severity diagnoses in the dataset.

**Fig. 2:**
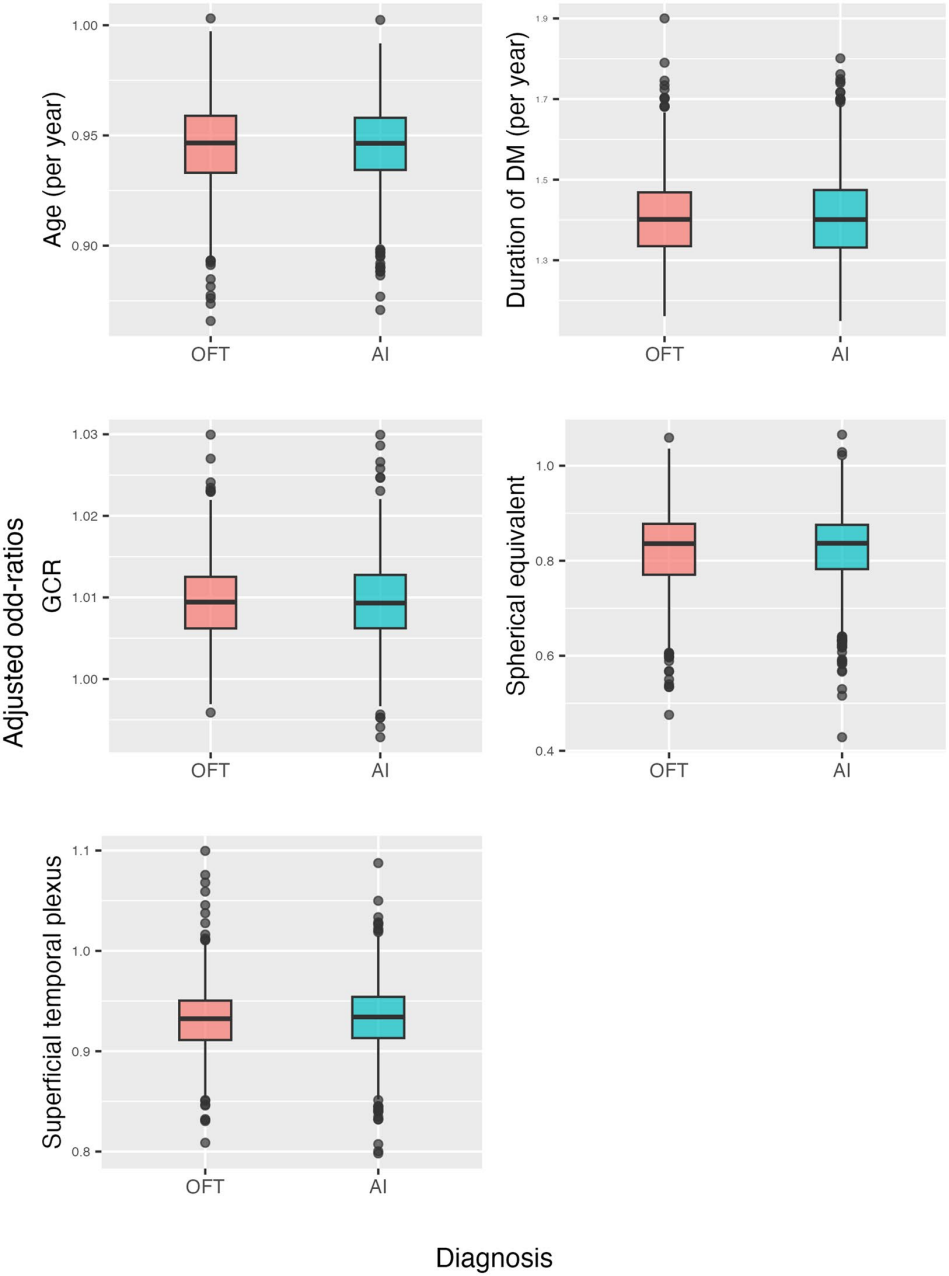
Sensitivity analysis. From the total sample (*n* = 498), 1,000 subsamples of size 300 were randomly drawn. Mixed logistic regression models were then fitted for each subsample, incorporating the covariates selected for both diagnostic methods. The accompanying box plots illustrate the distribution of the 1,000 estimated odds ratios for each covariate across the two diagnoses

Figure 2 presents the sensitivity analysis, in which 1000 random subsamples of size 300 were drawn from the total sample (*n* = 498). For each covariate, the distribution of the 1000 odds ratios is depicted using box plots.

Table 11 presents the intraclass correlation coefficients (ICC), used to assess the correlation between both eyes according to each diagnostic method. These coefficients were derived from the respective mixed-effects logistic models. For the OFT diagnosis, the estimated ICC was 0.912 (95% CI: 0.878–0.991), while for the AI diagnosis it was 0.801 (95% CI: 0.745–0.976).

**Table 11:** Intraclass correlation coefficients for to correlation between both eyes

	OFT	AI
Adjusted for the covariates in the model	0.912 (0.878 - 0.991)	*0.801 (0.745 - 0.976)*

Data are the ICCs (95% CI)

## Discussion

The detection of DR is a complex image-interpretation task and a key step in any successful screening program.

The findings of this study (Tables 2, 3, 4 and 5) reinforce the strong association between the progression of DR and alterations in key ocular parameters. Specifically, changes in visual acuity, DME, nuclear lens opacification, and reductions in vascular density exhibited a significant correlation with disease severity. These results provide further insights into the systemic impact of DR on ocular health and highlight the relevance of these parameters as potential indicators of disease progression. Conversely, cortical lens opacity did not show significant differences among the studied groups, suggesting that its progression may not be directly linked to DR severity. These findings underscore the importance of a comprehensive ophthalmological assessment in patients with DR, emphasizing the need for thorough monitoring to detect and manage disease-related complications effectively. These insights highlight the critical role of early detection and continuous monitoring using OCT to assess disease progression. Implementing OCT as a routine tool for evaluating structural and vascular alterations in DR could enhance clinical decision-making, facilitating timely therapeutic interventions aimed at preserving visual function and mitigating disease-associated complications. Overall, these findings contribute to a deeper understanding of the ophthalmic manifestations of DR and reinforce the importance of integrating multimodal imaging techniques into routine clinical practice. Further studies with larger cohorts and longitudinal designs are warranted to validate these associations and explore their potential implications for personalized disease management strategies [26, 30, 31, 38, 49, 50].

The analysis of sensitivity and specificity values highlights the robust diagnostic performance of the AI system in detecting DR. The high sensitivity observed suggests that the system is highly effective in identifying positive cases, reinforcing its utility as a screening tool for early disease detection. Similarly, the specificity is also high, although slightly lower than sensitivity, indicating a minor risk of overdiagnosis due to false-positive results. This could lead to unnecessary referrals for further ophthalmologic evaluation, which, while ensuring comprehensive patient care, may also contribute to an increased burden on healthcare systems. These high sensitivity and specificity values obtained are consistent with the results reported in other similar studies [51, 52].

The confidence intervals for both sensitivity and specificity demonstrate a high degree of reliability in these estimates, with slightly greater variability observed in specificity. This variability underscores the need for continuous refinement of AI algorithms to further optimize diagnostic accuracy and minimize unnecessary referrals. Nevertheless, these findings support the implementation of AI-assisted diagnostic methods as a valuable tool for DR detection, providing high sensitivity for disease identification and good specificity for distinguishing negative cases. The overall performance of the system reinforces its potential as an effective and reliable screening approach, particularly in large-scale screening programs aimed at early detection and timely intervention. Although EyeArt^®^ demonstrated high sensitivity, the moderately lower specificity implies a proportion of false positives, particularly in cases where severity was overestimated. From a clinical perspective, this may lead to increased referrals to ophthalmologists, which, while ensuring patient safety, may overload referral systems, especially in resource-limited settings. Such an increase in workload could affect waiting times and allocation of clinical resources, potentially reducing efficiency in managing truly severe cases. Hence, balancing sensitivity with specificity is essential to optimize screening workflows.

The multivariate logistic regression analysis identified several significant predictors of DR, including age, diabetes duration, DME, and CRT detected by OCT (CRT-OCT). These findings underscore the multifactorial nature of DR progression and highlight the importance of comprehensive patient management. In particular, glycemic control, blood pressure regulation, and consideration of disease duration should be key components in the clinical approach to diabetic patients to mitigate the risk of retinopathy development and progression.

The kappa coefficient values indicate a moderate level of agreement between diagnoses related to the severity of DR as determined by the ophthalmologist and the EyeArt^®^ AI system. However, when the agreement is assessed in the context of screening for a binary diagnosis—determining the presence or absence of DR without specifying its severity—the kappa coefficient approaches a value of one, indicating an almost perfect level of concordance between the ophthalmologist and the AI system. This high level of diagnostic accuracy supports the clinical and research applicability of AI-assisted methods, particularly in screening programs designed to differentiate between healthy individuals and those with DR. The strong agreement observed in both eyes further reinforces the reliability of this approach, highlighting its potential as an effective tool for large-scale DR detection and early intervention strategies. These results are consistent with other studies [51, 52]. However, this study demonstrates significantly higher concordance values compared to those of Wang et al. [53], Rajalakshmi et al. [54], Kim et al. [55], Mokhanshi et al. [56], and Cicinelli et al. [57]. This improvement in concordance is likely attributable to the refinement of the AI program and the quality of the images obtained with the device used.

In the scatter plot, it is evident that the AI program tends to overestimate the severity of DR as determined by the ophthalmologist. Notably, there are no data points below the bisector, indicating the absence of underdiagnosis cases. However, several points are located above the bisector, demonstrating that the EyeArt^®^ system has a tendency to overestimate the severity of DR compared to the ophthalmologist’s assessment. As previously noted, the degree of agreement, as measured by the Kappa coefficient, was higher (0.923 for right eyes and 0.949 for left eyes) when comparing the binary classification between the ophthalmologist and the AI, i.e., when the diagnosis was limited to determining the presence or absence of DR, which is the primary objective of screening programs. However, when the Kappa coefficient was calculated based on the severity grading of DR, the level of agreement was lower (0.650 for right eyes and 0.693 for left eyes). The scatter plot further confirms that the lower agreement observed in DR grading is due to the AI system’s tendency to overestimate disease severity.

The results of mixed-effects logistic refression models (Table 10) indicate that both age and spherical equivalent act as protective factors, whereas diabetes duration, CRT determined by OCT, and decreased vascular density in the temporal superficial plexus are significantly associated with a higher risk of DR in both models. These associations are consistent with previous literature [53] and reinforce the role of structural and functional retinal parameters in the progression of the disease. Although the AIC values were slightly higher in the AI-based model (1045.7 vs 957), indicating a marginally lower model fit, the similarity in the significant predictors suggests that EyeArt® effectively captures the key clinical determinants of DR identified by specialists, supporting its validity as a complementary diagnostic tool.

Table 10 presents the intraclass correlation coefficients (ICCs) assessing the concordance between both eyes for each diagnostic method. The high ICC observed for the ophthalmologist-based diagnosis (0.912; 95% CI: 0.878–0.991) indicates a strong inter-eye correlation, reflecting the expected bilateral nature of DR. Similarly, the AI-based model also demonstrated a substantial ICC (0.801; 95% CI: 0.745–0.976), confirming consistent diagnostic behavior across eyes. Although slightly lower than the ophthalmologist’s value, this result underscores the robust and reproducible performance of EyeArt® in detecting DR at the patient level, further supporting its reliability for bilateral screening applications.

Comparatively, other validated AI systems—such as IDx-DR®, Retmarker®, and RetinaLyze®—show similar or slightly lower diagnostic performance (Table 12). IDx-DR®, validated under FDA oversight, achieved 87.2% sensitivity and 90.7% specificity in primary care [57], while EyeArt® demonstrated superior sensitivity (100%) and comparable specificity [58]. Retmarker® has shown competitive accuracy in European screening networks, emphasizing scalability [59, 60], and RetinaLyze® has yielded more variable results across populations, with sensitivity ranging from 35.7% to 89% [61, 62]. These differences likely reflect variations in camera type, pupil dilation, and reference standards rather than fundamental algorithmic disparities.

**Table 12:** Comparative between different ai platforms or dr diagnosis

System	Study/Setting	Population (n)	Sensitivity % (95% CI)	Specificity % (95% CI)	Ungradable %
**EyeArt®**	Our study (Retisalud), regional screening	499	**100.0** (98.1–100)	**93.5** (90.2–96.0)	0.2
**IDxDR® (LumineticsCore™)** [1, 2]	Pivotal trial in 10 US primary care clinics	900	**87.2** (81.8–91.2)	**90.7** (88.3–92.7)	3.9
**IDxDR®** [3]	Teleophthalmology vs hybrid workflow	193	95.5 (86.7–100)	60.3 (47.7–72.9)	37.5
**IDxDR® vs RetCAD** [4]	Direct comparison, Poland multicenter	758	99.3 (–)	68.9 (–)	–
**RetinaLyze®** [5]	Pilot comparison vs IDxDR, Poland	170	74–90	72–94	–
**RetinaLyze®** [6]	Validation in Arab population (Saudi Arabia)	239	**35.7**	**83.3**	–
**Retmarker®** [7]	Reviewed in Modern Approach to DR Diagnostics	–	~90	~85	–

Several limitations of the study must be acknowledged. The study population was drawn from a single regional screening program (Retisalud, Canary Islands), which may limit the generalizability of results to other settings. The small number of proliferative DR cases (*n* = 2) precluded robust assessment in advanced disease stages, though this reflects the effectiveness of early detection in the local program. Additionally, the reference standard relied on grading by a single ophthalmologist, ensuring consistency but preventing evaluation of interobserver reliability. Future multicenter studies with more diverse populations and multiple graders are needed to confirm these findings and validate the algorithm in advanced DR.

Overall, this study supports the integration of EyeArt^®^ into real-world DR screening workflows. Its high sensitivity and strong agreement with ophthalmologists make it a reliable adjunct tool for early detection. Implementing hybrid workflows—where positive AI results are reviewed by trained graders—may further optimize efficiency and minimize unnecessary referrals, facilitating the safe adoption of autonomous AI systems in public health screening strategies.

From a clinical perspective, the implementation of autonomous AI systems such as EyeArt® could significantly improve the efficiency of DR screening programs. By rapidly identifying patients requiring specialist referral, these tools can optimize ophthalmologists’ workload and facilitate early intervention, particularly in areas with limited access to eye care services. Moreover, integrating AI-driven screening into existing public health infrastructures could enhance coverage, standardize diagnostic criteria, and ultimately contribute to reducing preventable vision loss at the population level.

Taken together, the findings of this study reinforce the clinical reliability of EyeArt® as an autonomous tool for DR screening in real-world conditions. Its high sensitivity and near-perfect agreement with ophthalmologists support its role as a safe first-line screening method capable of identifying patients at risk of vision-threatening disease. Although further validation in multicenter and demographically diverse populations is warranted, the integration of EyeArt® into organized screening programs could substantially enhance early detection rates, reduce diagnostic delays, and alleviate the burden on specialized ophthalmic services. By enabling accurate, scalable, and cost-effective screening, AI systems such as EyeArt® have the potential to transform diabetic eye care and contribute meaningfully to the prevention of avoidable blindness.

## Conclusions

The EyeArt® artificial intelligence system demonstrated excellent sensitivity and specificity for detecting DR, showing almost perfect agreement with ophthalmologists in binary diagnosis. Although the algorithm tended to overestimate disease severity, this behavior reflects a safety-oriented design that prioritizes sensitivity to minimize false negatives, an essential feature in screening programs. Despite the absence of an a priori sample size calculation, post-hoc analysis confirmed sufficient statistical power to support the robustness of the results.

These findings reinforce the potential of autonomous AI as a reliable adjunct for DR screening, enabling earlier detection, improved access to diagnosis, and optimized resource allocation in healthcare systems. Future multicenter studies involving larger and more diverse populations, multiple graders, and a broader spectrum of disease severity will be necessary to confirm generalizability and refine grading performance.

Integrating autonomous AI systems such as EyeArt® into organized national screening frameworks represents a transformative opportunity to expand equitable access to ophthalmic care, reduce diagnostic delays, and ultimately lower the global burden of preventable vision loss caused by diabetic retinopathy.

## Electronic supplementary material

Below is the link to the electronic supplementary material.

Supplementary Material 1

## Data Availability

No datasets were generated or analysed during the current study.
