# Supplementary material for: Evaluation of the degree of agreement in the diagnosis of diabetic retinopathy between ophthalmologists and EyeArt®
Source: Int J Retina Vitreous. 2025 Nov 19;11:125. doi: 10.1186/s40942-025-00748-4 (PMC12628637; doi:10.1186/s40942-025-00748-4)
Supplement: Supplementary file 1 — Supplementary Material 1 [file 40942_2025_748_MOESM1_ESM.docx]

### Univariate analysis

### Reading data

(za <- qnorm(.975))
setEPS(horizontal=FALSE,paper="special",onefile=FALSE)
library(readxl);library(writexl);library(openxlsx);library(pander)
library(glmmTMB);library(glmmLasso);library(lme4);library(irr);library(epiR)

library(ggplot2);library(gridExtra);library("ggpubr");library(ggrepel);library(ggimage)

panderOptions('table.split.table',250)
setwd("/Users/pedrosaavedra/Library/CloudStorage/OneDrive-UniversidaddeLasPalmasdeGranCanaria/Tesis/IsabelGuedes")
df <- read_xlsx("DiabeticRetinopathy_03102025.xlsx",1)
(n <- nrow(df))
(za <- qnorm(.975))
t.age <- quantile(df$Age,probs <- c(1/3,2/3))
at <- ifelse(df$Age<t.age[1],0,1)+ifelse(df$Age<t.age[2],0,1)
df$Age_T <- factor(at,levels=0:2,labels=c("< 62","62 - 71","> 71"))
boxplot(df$Age ~ df$Age_T)

df$Sex <- factor(df$Sex.male,levels=1:0,labels=c("Male","Female"))
df$AHT <- factor(df$HTA,levels=0:1,labels=c("Normotensive","Hypertensive"))

t.dm <- quantile(df$Years_DM ,probs=c(1/3,2/3))
tdm <- ifelse(df$Years_DM<t.dm[1],0,1)+ifelse(df$Years_DM<t.dm[2],0,1)
df$Years_DM_T <- factor(tdm,levels=0:2,labels=c("< 5","5 - 9","> 9"))
boxplot(df$Years_DM ~ df$Years_DM_T)

ds <- read_xlsx("DiabeticRetinopathy_03102025.xlsx",2)
(ns <- nrow(ds))

ds$RD.OFT <- factor(ds$Retinopathy.OFT,levels=0:3,
 labels=c("No","Mild nonproliferative",
 "Moderate nonproliferative",
 "Severe non-proliferative"))
table(ds$RD.OFT)

ds$RD.IA<- factor(ds$Retinopathy.IA,levels=0:4,
 labels=c("No","Mild nonproliferative",
 "Moderate nonproliferative",
 "Severe non-proliferative",
 "Proliferative"))
table(ds$RD.IA)
### Right eye
dr0 <- subset(ds,Eye=="R")
dr <- merge(df,dr0,by="ID")
nrow(dr)

### Left eye
dl0 <- subset(ds,Eye=="L")
dl <- merge(df,dl0,by="ID")
nrow(dl)

ds$Retinopathy.IA[ds$Retinopathy.IA==-1]=NA
table(ds$Retinopathy.IA)
ds$ret.dm <- ifelse(ds$Retinopathy.OFT==0,0,1)
table(ds$ret.dm)

ds$ret.dm.IA <- ifelse(ds$Retinopathy.IA==0,0,1)
table(ds$ret.dm.IA)

emd <- NULL
rd <- NULL
repe=NULL
for(i in 1:n)
{
 jj <- which((ds$ID==df$ID[i]))
 repe=c(repe,length(jj))
 emd <- c(emd,sum(ds$EMD.OCT[jj]))
 rd <- c(rd,sum(ds$ret.dm[jj]))
}
table(rd)
df$RD <- factor(rd,levels=0:2,labels=c("No","Unilateral","Bilateral"))
table(df$RD)

# df --> ds
age=NULL
for(j in 1:ns)
{
 nh <- ds$ID[j]
 jj <- which(df$ID==nh)
 age <- c(age,df$Age[j])
}

# df --\> ds = dfs
dfs <- merge(df,ds,by="ID")
dfs$id <- as.factor(dfs$ID)

### Data train for the LASSO (no funciona bien con valores perdidos)
attach(dfs)
nrow(dfs)
dt <- na.omit(data.frame(id,ret.dm,ret.dm.IA,Age,DM1,DM2,EMD.OCT,GCR.OCT,Spherical.equivalent,
 VD_A.OCT.SUPERFICIAL.PLEXUS.TEMPORAL,VD_A.OCT.SUPERFICIAL.PLEXUS.NASAL,
 Crystalline.N,Years_DM))
nrow(dt)

### Both eyes
rd.r <- ifelse(dr0$RD.OFT=="No",0,1)
rd.l <- ifelse(dl0$RD.OFT=="No",0,1)
dd <- data.frame(ID=dr0$ID,rd.r)
di <- data.frame(ID=dl0$ID,rd.l)
dboth <- merge(dd,di,by="ID")

(tb <- table(dboth$rd.r,rd.l))

ret.dm.OFT_12 <- factor(2-ret.dm,levels=1:2,labels=c("Yes","No"))
ret.dm.IA_12 <- factor(2-ret.dm.IA,levels=1:2,labels=c("Yes","No"))

# Right eye
(tr.bin <- table(ret.dm.IA_12[Eye=="R"],ret.dm.OFT_12[Eye=="R"]))
(kappa.bin.R <- epi.kappa(tb, method = "cohen", alternative = c("two.sided"), conf.level = 0.95))

# TABLE 1. Patient characteristics: overall and according to RD

with(df,{
 X1=data.frame(Age)
 X2=data.frame(Sex.male,HTA,DM2,DM1,DLP,Asma,IAM,EPOC,IC,IRC,SAOS)
 X3=data.frame(Sex.male)
 X4=data.frame(Years_DM)
 Tabla(X1,X2,X3,X4, RD)
})

g <- dr$Retinopathy.OFT

# TABLE 2. Eyes characteristics

with(dr,{
 X1=data.frame(GCR.OCT)
 X2=data.frame(EMD.OCT,EDEMA.MACULAR.IA)
 X3=data.frame(Crystalline.C,Crystalline.P)
 X4=data.frame(AVMC,Spherical.equivalent,Crystalline.N,GCR.OCT,
 VD_A.OCT.SUPERFICIAL.PLEXUS.CENTRAL,
 VD_A.OCT.SUPERFICIAL.PLEXUS.SUPERIOR,
 VD_A.OCT.SUPERFICIAL.PLEXUS.TEMPORAL,
 VD_A.OCT.SUPERFICIAL.PLEXUS.INFERIOR,
 VD_A.OCT.SUPERFICIAL.PLEXUS.NASAL)
 Tabla(X1,X2,X3,X4, RD.OFT)
})

with(dl,{
 X1=data.frame(GCR.OCT)
 X2=data.frame(EMD.OCT,EDEMA.MACULAR.IA)
 X3=data.frame(Crystalline.C,Crystalline.P)
 X4=data.frame(AVMC,Spherical.equivalent,Crystalline.N,GCR.OCT,
 VD_A.OCT.SUPERFICIAL.PLEXUS.CENTRAL,
 VD_A.OCT.SUPERFICIAL.PLEXUS.SUPERIOR,
 VD_A.OCT.SUPERFICIAL.PLEXUS.TEMPORAL,
 VD_A.OCT.SUPERFICIAL.PLEXUS.INFERIOR,
 VD_A.OCT.SUPERFICIAL.PLEXUS.NASAL)
 #Tabla(X1,X2,X3,X4, RD.OFT)
 Tabla(X1,X2,X3,X4, RD.IA)
})

### Correlations

dd <- data.frame(ID=ds$ID[Eye=="R"],RD.OCT.R=ds$Retinopathy.OFT[Eye=="R"],RD.IA.R=ds$Retinopathy.IA[Eye=="R"])
di <- data.frame(ID=ds$ID[Eye=="L"],RD.OCT.L=ds$Retinopathy.OFT[Eye=="L"],RD.IA.L=ds$Retinopathy.IA[Eye=="L"])
ddi <- merge(dd,di,by="ID")
nrow(ddi)
str(ddi)

### Correlación OCT
d.OCT <- ifelse(ddi$RD.OCT.R==0,0,1)
i.OCT <- ifelse(ddi$RD.OCT.L==0,0,1)
(t.OCT <- table(d.OCT,i.OCT))
100*sum(diag(t.OCT))/sum(t.OCT)


### Correlación IA
d.IA <- ifelse(ddi$RD.IA.R==0,0,1)
i.IA <- ifelse(ddi$RD.IA.L==0,0,1)
(t.IA <- table(d.IA,i.IA))
100*sum(diag(t.IA))/sum(t.IA)

### Diabetic retinopathy by OFT

Z <- cbind(Age,Years_DM,GCR.OCT,Spherical.equivalent,VD_A.OCT.SUPERFICIAL.PLEXUS.TEMPORAL)
dt <- data.frame(id,ret.dm,Z)
nrow(dt)

## [1] 996

model_oct <- glmmTMB(ret.dm ~ Z + (1 | id),
 data = dt,family = binomial(link = "logit"))
(sm <- summary(model_oct))

## Family: binomial ( logit )
## Formula: ret.dm ~ Z + (1 | id)
## Data: dt
##
## AIC BIC logLik deviance df.resid
## 957.0 991.3 -471.5 943.0 989
##
## Random effects:
##
## Conditional model:
## Groups Name Variance Std.Dev.
## id (Intercept) 34.26 5.853
## Number of obs: 996, groups: id, 498
##
## Conditional model:
## Estimate Std. Error z value Pr(>|z|)
## (Intercept) -1.748362 3.050813 -0.573 0.56659
## ZAge -0.082091 0.030353 -2.705 0.00684 **
## ZYears_DM 0.460380 0.081189 5.670 1.42e-08 ***
## ZGCR.OCT 0.013067 0.006303 2.073 0.03816 *
## ZSpherical.equivalent -0.175119 0.085408 -2.050 0.04033 *
## ZVD_A.OCT.SUPERFICIAL.PLEXUS.TEMPORAL -0.074875 0.037816 -1.980 0.04770 *
## ---
## Signif. codes: 0 '***' 0.001 '**' 0.01 '*' 0.05 '.' 0.1 ' ' 1

tab <- sm$coeff$cond
b <- tab[,1]
sb <- tab[,2]
pval <- ifelse(tab[,4]<.001,"< 0.001",round(tab[,4],3))
(sm$AICtab[1])

## AIC
## 956.9543

OR=exp(b);OR.l=exp(b-za*sb);OR.u=exp(b+za*sb)
IC95=array(sprintf("%.3f (%.3f %.3s %.3f)",OR,OR.l,"; ",OR.u),dim=c(length(OR),1))


p_lrt=NULL;aic=NULL
for(j in 1:ncol(Z))
{
 ml <- glmmTMB(ret.dm ~ Z[,-j] + (1 | id),
 data = dt,family = binomial(link = "logit"))
 sm2 <- summary(ml)
 aov <- anova(model_oct,ml,test="Chisq")
 pv=ifelse(aov$"Pr(>Chisq)"[2]<0.001,"< 0.001",round(aov$"Pr(>Chisq)"[2],3))
 p_lrt=c(p_lrt,pv)
 aic=c(aic,sm2$AICtab[1])
}

pval=c(0,p_lrt)
AIC=c(sm$AICtab[1],aic)
tb=array(sprintf("%.3f (%.3f)",b,sb),dim=c(ncol(Z)+1,1))
tf=cbind(tb,pval,round(AIC,1),IC95)
lb <- attr(tab,"dimnames")[[1]]
row.names(tf) <- lb
colnames(tf) <- c("Coefficient (SE)","P-value","AIC","Odd-ratio (95% CI)")
pander(tf)

var_cor <- VarCorr(model_oct)
(sigma_u_sq <- var_cor$cond$id[1,1])

## [1] 34.26075

std_dev <- sqrt(sigma_u_sq)

# Calcular ICC
icc <- sigma_u_sq / (sigma_u_sq + (pi^2)/3)

# Imprimir resultados
cat("Varianza de los efectos aleatorios:", sigma_u_sq, "\n")

## Varianza de los efectos aleatorios: 34.26075

cat("Desviación estándar:", std_dev, "\n")

## Desviación estándar: 5.853268

cat("ICC:", icc, "\n")

## ICC: 0.9123884

model_oct <- glmmTMB(ret.dm ~ 1 + (1 | id),
 data = dt,family = binomial(link = "logit"))
(sm <- summary(model_oct))

## Family: binomial ( logit )
## Formula: ret.dm ~ 1 + (1 | id)
## Data: dt
##
## AIC BIC logLik deviance df.resid
## 968.1 977.9 -482.1 964.1 994
##
## Random effects:
##
## Conditional model:
## Groups Name Variance Std.Dev.
## id (Intercept) 169 13
## Number of obs: 996, groups: id, 498
##
## Conditional model:
## Estimate Std. Error z value Pr(>|z|)
## (Intercept) -7.7892 0.5165 -15.08 <2e-16 ***
## ---
## Signif. codes: 0 '***' 0.001 '**' 0.01 '*' 0.05 '.' 0.1 ' ' 1

### Diabetic retinopathy by AI

Z <- cbind(Age,Years_DM,DM1,GCR.OCT,Spherical.equivalent,VD_A.OCT.SUPERFICIAL.PLEXUS.TEMPORAL)
dt <- data.frame(id,ret.dm.IA,Z)
nrow(dt)

## [1] 996

model_ai <- glmmTMB(ret.dm.IA ~ Z + (1 | id),
 data = dt,family = binomial(link = "logit"))
(sm <- summary(model_ai))

## Family: binomial ( logit )
## Formula: ret.dm.IA ~ Z + (1 | id)
## Data: dt
##
## AIC BIC logLik deviance df.resid
## 1045.6 1084.9 -514.8 1029.6 988
##
## Random effects:
##
## Conditional model:
## Groups Name Variance Std.Dev.
## id (Intercept) 13.7 3.701
## Number of obs: 996, groups: id, 498
##
## Conditional model:
## Estimate Std. Error z value Pr(>|z|)
## (Intercept) -1.024155 2.447960 -0.418 0.6757
## ZAge -0.043064 0.022270 -1.934 0.0531 .
## ZYears_DM 0.331817 0.065840 5.040 4.66e-07 ***
## ZDM1 2.218348 1.580004 1.404 0.1603
## ZGCR.OCT 0.009652 0.004799 2.011 0.0443 *
## ZSpherical.equivalent -0.179676 0.073487 -2.445 0.0145 *
## ZVD_A.OCT.SUPERFICIAL.PLEXUS.TEMPORAL -0.068368 0.030277 -2.258 0.0239 *
## ---
## Signif. codes: 0 '***' 0.001 '**' 0.01 '*' 0.05 '.' 0.1 ' ' 1

(tab <- sm$coeff$cond)

## Estimate Std. Error z value
## (Intercept) -1.024155309 2.447960387 -0.4183709
## ZAge -0.043063892 0.022270051 -1.9337133
## ZYears_DM 0.331817420 0.065839724 5.0397754
## ZDM1 2.218347551 1.580004387 1.4040135
## ZGCR.OCT 0.009651592 0.004798517 2.0113697
## ZSpherical.equivalent -0.179676150 0.073486846 -2.4450110
## ZVD_A.OCT.SUPERFICIAL.PLEXUS.TEMPORAL -0.068368168 0.030276637 -2.2581163
## Pr(>|z|)
## (Intercept) 6.756760e-01
## ZAge 5.314839e-02
## ZYears_DM 4.660786e-07
## ZDM1 1.603148e-01
## ZGCR.OCT 4.428642e-02
## ZSpherical.equivalent 1.448477e-02
## ZVD_A.OCT.SUPERFICIAL.PLEXUS.TEMPORAL 2.393841e-02

b <- tab[,1]
sb <- tab[,2]
pval <- ifelse(tab[,4]<.001,"< 0.001",round(tab[,4],3))
(sm$AICtab[1])

## AIC
## 1045.645

OR=exp(b);OR.l=exp(b-za*sb);OR.u=exp(b+za*sb)
IC95=array(sprintf("%.3f (%.3f %.3s %.3f)",OR,OR.l,"; ",OR.u),dim=c(length(OR),1))


p_lrt=NULL;aic=NULL
for(j in 1:ncol(Z))
{
 ml <- glmmTMB(ret.dm.IA ~ Z[,-j] + (1 | id),
 data = dt,family = binomial(link = "logit"))
 sm2 <- summary(ml)
 aov <- anova(model_ai,ml,test="Chisq")
 pv=ifelse(aov$"Pr(>Chisq)"[2]<0.001,"< 0.001",round(aov$"Pr(>Chisq)"[2],3))
 p_lrt=c(p_lrt,pv)
 aic=c(aic,sm2$AICtab[1])
}

pval=c(0,p_lrt)
AIC=c(sm$AICtab[1],aic)
tb=array(sprintf("%.3f (%.3f)",b,sb),dim=c(ncol(Z)+1,1))
tf=cbind(tb,pval,round(AIC,1),IC95)
lb <- attr(tab,"dimnames")[[1]]
row.names(tf) <- lb
colnames(tf) <- c("Coefficient (SE)","P-value","AIC","Odd-ratio (95% CI)")
pander(tf)

var_cor <- VarCorr(model_ai)
(sigma_u_sq <- var_cor$cond$id[1,1])

## [1] 13.6995

std_dev <- sqrt(sigma_u_sq)

# Calcular ICC
icc <- sigma_u_sq / (sigma_u_sq + (pi^2)/3)

# Imprimir resultados
cat("Varianza de los efectos aleatorios:", sigma_u_sq, "\n")

## Varianza de los efectos aleatorios: 13.6995

cat("Desviación estándar:", std_dev, "\n")

## Desviación estándar: 3.701284

cat("ICC:", icc, "\n")

## ICC: 0.8063573

# Figure

lb <- c("No","Mild","Moderate","Severe","Proliferative")
gf <- list()

qr <- "Right eyes"
OFT.R <- as.factor(Retinopathy.OFT[Eye=="R"])
IA.R <- as.factor(Retinopathy.IA[Eye=="R"])
dz <- data.frame(OFT.R,IA.R,qr)
gf[[1]] <- ggplot(dz,aes(x=OFT.R,y=IA.R))+
 geom_jitter(width = 0.1,height=0.1,color="blue",size=0.5)+
 labs(x="",y="Diagnosis by AI")+
 facet_grid(~qr)+
 scale_x_discrete(breaks=c("0","1","2","3","4"),labels=lb)+
 scale_y_discrete(breaks=c("0","1","2","3","4"),labels=lb)

ql <- "Left eyes"
OFT.L <- as.factor(Retinopathy.OFT[Eye=="L"])
IA.L <- as.factor(Retinopathy.IA[Eye=="L"])
dz <- na.omit(data.frame(OFT.L,IA.L,ql))
gf[[2]] <- ggplot(dz,aes(x=OFT.L,y=IA.L))+
 geom_jitter(width = 0.1,height=0.1,color="red",size=0.5)+
 labs(x="",y="")+
 facet_grid(~ql)+
 scale_x_discrete(breaks=c("0","1","2","3","4"),labels=lb)+
 scale_y_discrete(breaks=c("0","1","2","3","4"),labels=lb)

table(IA.L)

## IA.L
## 0 1 2 3 4
## 336 51 65 43 3

fig <- ggarrange(gf[[1]],gf[[2]],nrow=1,ncol=2)
f1 <- annotate_figure(fig,top=text_grob("Diagnosis of diabetic retinopathy"),
 bottom=text_grob("Diagnosis by OFT",size=12))
ggsave(file = "RD.tiff", print(f1),height=5,width=8)

Retinopathy.OFT.R=Retinopathy.OFT[Eye=="R"]
Retinopathy.IA.R = Retinopathy.IA[Eye=="R"]
diag <- cbind(Retinopathy.OFT.R,Retinopathy.IA.R)
kappa2(diag, "squared")
kappam.fleiss(diag)

# Kappa

lb <- c("No","Mild","Moderate","Severe","Proliferative")
# right eye
Retinopathy.OFT.R=factor(Retinopathy.OFT[Eye=="R"],levels=0:4,labels=lb)
Retinopathy.IA.R = factor(Retinopathy.IA[Eye=="R"],levels=0:4,labels=lb)
(tr <- table(Retinopathy.OFT.R,Retinopathy.IA.R))

## Retinopathy.IA.R
## Retinopathy.OFT.R No Mild Moderate Severe Proliferative
## No 324 9 5 2 1
## Mild 0 48 44 5 0
## Moderate 0 0 21 23 1
## Severe 0 0 0 15 0
## Proliferative 0 0 0 0 0

(kappa.R <- epi.kappa(tr, method = "fleiss", alternative = c("two.sided"), conf.level = 0.95))

## $prop.agree
## obs exp
## 1 0.8192771 0.4832099
##
## $pabak
## est lower upper
## 1 0.6385542 0.5652274 0.7041937
##
## $kappa
## est se lower upper
## 1 0.6502973 0.02897499 0.5935073 0.7070872
##
## $z
## test.statistic p.value
## 1 22.4434 1.484593e-111

k.R1 <- kappa.R[[1]]
k.R3 <- kappa.R[[3]]
tr <- array(c(sprintf("%.3f",k.R1[1]),sprintf("%.3f",k.R1[2]),sprintf("%.3f (%.3f; %.3f)",k.R3[1],k.R3[3],k.R3[4])),dim=c(1,3))

# left eye
Retinopathy.OFT.L=factor(Retinopathy.OFT[Eye=="L"],levels=0:4,labels=lb)
Retinopathy.IA.L = factor(Retinopathy.IA[Eye=="L"],levels=0:4,labels=lb)
(tl <- table(Retinopathy.OFT.L,Retinopathy.IA.L))

## Retinopathy.IA.L
## Retinopathy.OFT.L No Mild Moderate Severe Proliferative
## No 336 7 3 1 0
## Mild 0 44 37 3 0
## Moderate 0 0 25 22 0
## Severe 0 0 0 17 3
## Proliferative 0 0 0 0 0

(kappa.L <- epi.kappa(tl, method = "fleiss", alternative = c("two.sided"), conf.level = 0.95))

## $prop.agree
## obs exp
## 1 0.8473896 0.5031814
##
## $pabak
## est lower upper
## 1 0.6947791 0.6254989 0.7556738
##
## $kappa
## est se lower upper
## 1 0.6928246 0.02904763 0.6358923 0.7497569
##
## $z
## test.statistic p.value
## 1 23.85133 9.810532e-126

k.L1 <- kappa.L[[1]]
k.L3 <- kappa.L[[3]]
tl <- array(c(sprintf("%.3f",k.L1[1]),sprintf("%.3f",k.L1[2]),sprintf("%.3f (%.3f; %.3f)",k.L3[1],k.L3[3],k.L3[4])),dim=c(1,3))

tab <- rbind(tr,tl)
row.names(tab) <- c("Right eye","Left eye")
colnames(tab) <- c("Observed","Expected","kappa (95%CI")
pander(tab)

|  | Observed | Expected | kappa (95%CI |
| --- | --- | --- | --- |
| **Right eye** | 0.819 | 0.483 | 0.650 (0.594; 0.707) |
| **Left eye** | 0.847 | 0.503 | 0.693 (0.636; 0.750) |

### Binocular sensitivities and specificities

library(caret)

dleft <- subset(ds,Eye=="L")
nrow(dleft)

## [1] 498

dright <- subset(ds,Eye=="R")
nrow(dright)

## [1] 498

dd <- merge(dright,dleft,by="ID")
detach()
attach(dd)

## The following object is masked from dfs:
##
## ID

r.OFT.R <- ifelse(Retinopathy.OFT.x==0,0,1)
r.OFT.L <- ifelse(Retinopathy.OFT.y==0,0,1)
r.IA.R <- ifelse(Retinopathy.IA.x==0,0,1)
r.IA.L <- ifelse(Retinopathy.IA.y==0,0,1)
table(Retinopathy.OFT.y,r.OFT.L)

## r.OFT.L
## Retinopathy.OFT.y 0 1
## 0 347 0
## 1 0 84
## 2 0 47
## 3 0 20

RD.OFT=ifelse(r.OFT.R+r.OFT.L==0,2,1)
RD.IA=ifelse(r.IA.R+r.IA.L==0,2,1)
xtable <- table(RD.IA,RD.OFT)
xtable

## RD.OFT
## RD.IA 1 2
## 1 189 20
## 2 0 289

rval <- epi.tests(xtable, method = "exact", digits = 3,conf.level = 0.95)
rval

## Outcome + Outcome - Total
## Test + 189 20 209
## Test - 0 289 289
## Total 189 309 498
##
## Point estimates and 95% CIs:
## --------------------------------------------------------------
## Apparent prevalence * 0.420 (0.376, 0.464)
## True prevalence * 0.380 (0.337, 0.424)
## Sensitivity * 1.000 (0.981, 1.000)
## Specificity * 0.935 (0.902, 0.960)
## Positive predictive value * 0.904 (0.856, 0.941)
## Negative predictive value * 1.000 (0.987, 1.000)
## Positive likelihood ratio 15.450 (10.112, 23.605)
## Negative likelihood ratio 0.000 (0.000, NaN)
## False T+ proportion for true D- * 0.065 (0.040, 0.098)
## False T- proportion for true D+ * 0.000 (0.000, 0.019)
## False T+ proportion for T+ * 0.096 (0.059, 0.144)
## False T- proportion for T- * 0.000 (0.000, 0.013)
## Correctly classified proportion * 0.960 (0.939, 0.975)
## --------------------------------------------------------------
## * Exact CIs

RD2.OFT=ifelse(r.OFT.R+r.OFT.L==0,1,2)
RD2.IA=ifelse(r.IA.R+r.IA.L==0,1,2)
xtable2 <- table(RD2.IA,RD2.OFT)
xtable2

## RD2.OFT
## RD2.IA 1 2
## 1 289 0
## 2 20 189

1. Determinar la Sensibilidad y Especificidad del programa de IA (frente al diagnóstico del Oftalmólogo que es el gold standard). Hay estudios en los que ya se ha publicado la S y E del EyeNuk que es el programa de IA que hemos empleado y de hecho se postulan que pueden haber diferencias en base a la etnia, edad y sexo.
2. Valorar la congruencia (si no recuerdo mal se realizaba a través del coeficiente Kappa) entre el diagnóstico realizado por el programa de IA y el oftalmólogo. La finalidad de esta determinación es poder valorar a posteriori si se podría establecer el programa de IA como cribado de la Retinopatía Diabética (RD) si existe un alto grado de congruencia entre ambos.

## Agreement (Y/N)

ret.dm.OFT_12 <- factor(2-ret.dm,levels=1:2,labels=c("Yes","No"))
ret.dm.IA_12 <- factor(2-ret.dm.IA,levels=1:2,labels=c("Yes","No"))

# Right eye
(tr.bin <- table(ret.dm.IA_12[Eye=="R"],ret.dm.OFT_12[Eye=="R"]))

##
## Yes No
## Yes 157 17
## No 0 324

(kappa.bin.R <- epi.kappa(tr.bin, method = "cohen", alternative = c("two.sided"), conf.level = 0.95))

## $prop.agree
## obs exp
## 1 0.9658635 0.5556443
##
## $pindex
## est se lower upper
## 1 -0.3353414 0.02983187 -0.3938108 -0.276872
##
## $bindex
## est se lower upper
## 1 0.03413655 0.02983187 -0.02433285 0.09260594
##
## $pabak
## est lower upper
## 1 0.9317269 0.891811 0.9599817
##
## $kappa
## est se lower upper
## 1 0.9231774 0.01831143 0.8872877 0.9590672
##
## $z
## test.statistic p.value
## 1 50.41537 0
##
## $mcnemar
## test.statistic df p.value
## 1 17 1 3.737982e-05

epi.tests(tr.bin, method = "exact", digits = 3, conf.level = 0.95)

## Outcome + Outcome - Total
## Test + 157 17 174
## Test - 0 324 324
## Total 157 341 498
##
## Point estimates and 95% CIs:
## --------------------------------------------------------------
## Apparent prevalence * 0.349 (0.308, 0.393)
## True prevalence * 0.315 (0.275, 0.358)
## Sensitivity * 1.000 (0.977, 1.000)
## Specificity * 0.950 (0.921, 0.971)
## Positive predictive value * 0.902 (0.848, 0.942)
## Negative predictive value * 1.000 (0.989, 1.000)
## Positive likelihood ratio 20.059 (12.620, 31.882)
## Negative likelihood ratio 0.000 (0.000, NaN)
## False T+ proportion for true D- * 0.050 (0.029, 0.079)
## False T- proportion for true D+ * 0.000 (0.000, 0.023)
## False T+ proportion for T+ * 0.098 (0.058, 0.152)
## False T- proportion for T- * 0.000 (0.000, 0.011)
## Correctly classified proportion * 0.966 (0.946, 0.980)
## --------------------------------------------------------------
## * Exact CIs

# Left eye
(tl.bin <- table(ret.dm.IA_12[Eye=="L"],ret.dm.OFT_12[Eye=="L"]))

##
## Yes No
## Yes 151 11
## No 0 336

(kappa.bin.L <- epi.kappa(tl.bin, method = "cohen", alternative = c("two.sided"), conf.level = 0.95))

## $prop.agree
## obs exp
## 1 0.9779116 0.568757
##
## $pindex
## est se lower upper
## 1 -0.3714859 0.02941038 -0.4291292 -0.3138427
##
## $bindex
## est se lower upper
## 1 0.02208835 0.02941038 -0.03555492 0.07973163
##
## $pabak
## est lower upper
## 1 0.9558233 0.9216442 0.9778464
##
## $kappa
## est se lower upper
## 1 0.9487798 0.01527196 0.9188473 0.9787123
##
## $z
## test.statistic p.value
## 1 62.12562 0
##
## $mcnemar
## test.statistic df p.value
## 1 11 1 0.0009111189

epi.tests(tl.bin, method = "exact", digits = 3, conf.level = 0.95)

## Outcome + Outcome - Total
## Test + 151 11 162
## Test - 0 336 336
## Total 151 347 498
##
## Point estimates and 95% CIs:
## --------------------------------------------------------------
## Apparent prevalence * 0.325 (0.284, 0.368)
## True prevalence * 0.303 (0.263, 0.346)
## Sensitivity * 1.000 (0.976, 1.000)
## Specificity * 0.968 (0.944, 0.984)
## Positive predictive value * 0.932 (0.882, 0.966)
## Negative predictive value * 1.000 (0.989, 1.000)
## Positive likelihood ratio 31.545 (17.636, 56.426)
## Negative likelihood ratio 0.000 (0.000, NaN)
## False T+ proportion for true D- * 0.032 (0.016, 0.056)
## False T- proportion for true D+ * 0.000 (0.000, 0.024)
## False T+ proportion for T+ * 0.068 (0.034, 0.118)
## False T- proportion for T- * 0.000 (0.000, 0.011)
## Correctly classified proportion * 0.978 (0.961, 0.989)
## --------------------------------------------------------------
## * Exact CIs

(tr <- table(Retinopathy.OFT.R,Retinopathy.IA.R))

## Retinopathy.IA.R
## Retinopathy.OFT.R No Mild Moderate Severe Proliferative
## No 324 9 5 2 1
## Mild 0 48 44 5 0
## Moderate 0 0 21 23 1
## Severe 0 0 0 15 0
## Proliferative 0 0 0 0 0

(Po <- sum(diag(tr))/sum(tr))

## [1] 0.8192771

er <- chisq.test(tr)$expected

## Warning in chisq.test(tr): Chi-squared approximation may be incorrect

(Pe <- sum(diag(er))/sum(er))

## [1] 0.4832099

(kappa.Cohen <- (Po-Pe)/(1-Pe))

## [1] 0.6502973

1. Valorar el grado de congruencia entre la determinación de Edema Macular Diabético (EMD) realizado por el programa de IA y el hallazgo de la OCT (que es el gold standard para el diagnóstico de EMD). De esta forma podríamos determinar si la IA es útil para valorar si existe o no EMD y en un posible programa de screening que aquellos pacientes que tengan EMD tengan prioridad en la valoración oftalmológica ya que son aquellos que requieren un tratamiento de forma precoz.

### Edema macular (Gold standard: OFT)

em.oft <- factor(2-EMD.OCT,levels=1:2,labels=c("Yes","No"))
em.ia <- factor(2-EDEMA.MACULAR.IA,levels=1:2,labels=c("Yes","No"))

## Right eye
tb.r <- table(em.ia[Eye=="R"],em.oft[Eye=="R"])
epi.tests(tb.r, method = "exact", digits = 3, conf.level = 0.95)

## Outcome + Outcome - Total
## Test + 13 7 20
## Test - 3 474 477
## Total 16 481 497
##
## Point estimates and 95% CIs:
## --------------------------------------------------------------
## Apparent prevalence * 0.040 (0.025, 0.061)
## True prevalence * 0.032 (0.019, 0.052)
## Sensitivity * 0.812 (0.544, 0.960)
## Specificity * 0.985 (0.970, 0.994)
## Positive predictive value * 0.650 (0.408, 0.846)
## Negative predictive value * 0.994 (0.982, 0.999)
## Positive likelihood ratio 55.830 (25.795, 120.839)
## Negative likelihood ratio 0.190 (0.069, 0.528)
## False T+ proportion for true D- * 0.015 (0.006, 0.030)
## False T- proportion for true D+ * 0.188 (0.040, 0.456)
## False T+ proportion for T+ * 0.350 (0.154, 0.592)
## False T- proportion for T- * 0.006 (0.001, 0.018)
## Correctly classified proportion * 0.980 (0.963, 0.990)
## --------------------------------------------------------------
## * Exact CIs

## left eye
tb.l <- table(em.ia[Eye=="L"],em.oft[Eye=="L"])
epi.tests(tb.l, method = "exact", digits = 3, conf.level = 0.95)

## Outcome + Outcome - Total
## Test + 18 8 26
## Test - 4 468 472
## Total 22 476 498
##
## Point estimates and 95% CIs:
## --------------------------------------------------------------
## Apparent prevalence * 0.052 (0.034, 0.076)
## True prevalence * 0.044 (0.028, 0.066)
## Sensitivity * 0.818 (0.597, 0.948)
## Specificity * 0.983 (0.967, 0.993)
## Positive predictive value * 0.692 (0.482, 0.857)
## Negative predictive value * 0.992 (0.978, 0.998)
## Positive likelihood ratio 48.682 (23.820, 99.493)
## Negative likelihood ratio 0.185 (0.076, 0.449)
## False T+ proportion for true D- * 0.017 (0.007, 0.033)
## False T- proportion for true D+ * 0.182 (0.052, 0.403)
## False T+ proportion for T+ * 0.308 (0.143, 0.518)
## False T- proportion for T- * 0.008 (0.002, 0.022)
## Correctly classified proportion * 0.976 (0.958, 0.987)
## --------------------------------------------------------------
## * Exact CIs

## Lab

1. Ver si existe relación entre los valores de angio-OCT (A-OCT) y el grado de RD, tiempo de evolución de la diabetes, edad, sexo y otras patologías que implican aumento del riesgo cardiovascular. La angio-oct nos da valores de densidad vascular de la capa profunda de la Retina. En la base de datos se recogieron valores a nivel macular central, superior, temporal, inferior y nasal. Como sabemos, la DM es una patología en la que se produce un daño de los vasos. Con este análisis lo que se pretende es saber si existen diferencias en la densidad vascular de aquellos pacientes con mayor grado de RD, mayor tiempo de evolución y la relación con otras comorbilidades recogidas en la tabla.

tab.AC <- function(ojo,g)
{
 detach()
 if(ojo=="Right") attach(dr) else attach(dl)
 Y=cbind(VD_A.OCT.SUPERFICIAL.PLEXUS.CENTRAL,VD_A.OCT.SUPERFICIAL.PLEXUS.SUPERIOR,
 VD_A.OCT.SUPERFICIAL.PLEXUS.TEMPORAL,VD_A.OCT.SUPERFICIAL.PLEXUS.INFERIOR,
 VD_A.OCT.SUPERFICIAL.PLEXUS.NASAL)
 nc <- length(table(g))
 tb1=NULL
 for(j in 1:ncol(Y))
 {
 my <- as.matrix(by(Y[,j],g,quantile,probs=c(.5,.25,.75),na.rm=TRUE))
 pv=round(kruskal.test(Y[,j] ~ g)$p.value,3)
 pval=c(ifelse(pv < 0.001,"< 0.001",round(pv,3)))
 tb2=NULL
 for(k in 1:nc)
 tb2=c(tb2,array(sprintf("%.1f (%.1f; %.1f)",my[[k]][1],my[[k]][2],my[[k]][3]),dim=c(1,1)))
 tb1=rbind(tb1,c(tb2,pval))
 }
 row.names(tb1)=colnames(Y)
 colnames(tb1) <- c(names(table(g)),"P-value")
 pander(paste("Eye = ",ojo))
 pander(tb1)
}
